# Embryo selection through artificial intelligence versus embryologists: a systematic review

**DOI:** 10.1093/hropen/hoad031

**Published:** 2023-08-15

**Authors:** M Salih, C Austin, R R Warty, C Tiktin, D L Rolnik, M Momeni, H Rezatofighi, S Reddy, V Smith, B Vollenhoven, F Horta

**Affiliations:** Department of Obstetrics and Gynaecology, Monash University, Clayton, Victoria, Australia; Department of Data Science and Artificial Intelligence, Faculty of Information Technology, Monash University, Clayton, Victoria, Australia; Department of Obstetrics and Gynaecology, Monash University, Clayton, Victoria, Australia; Department of Data Science and Artificial Intelligence, Faculty of Information Technology, Monash University, Clayton, Victoria, Australia; Department of Obstetrics and Gynaecology, Monash University, Clayton, Victoria, Australia; School of Engineering, RMIT University, Melbourne, Victoria, Australia; Department of Obstetrics and Gynaecology, Monash University, Clayton, Victoria, Australia; Women’s and Newborn Program, Monash Health, Melbourne, Victoria, Australia; Department of Obstetrics and Gynaecology, Monash University, Clayton, Victoria, Australia; Department of Data Science and Artificial Intelligence, Faculty of Information Technology, Monash University, Clayton, Victoria, Australia; Monash Data Future Institute, Monash University, Clayton, Victoria, Australia; School of Medicine, Deakin University, Geelong, Victoria, Australia; Department of Obstetrics and Gynaecology, Monash University, Clayton, Victoria, Australia; Department of Obstetrics and Gynaecology, Monash University, Clayton, Victoria, Australia; Women’s and Newborn Program, Monash Health, Melbourne, Victoria, Australia; Monash IVF, Melbourne, Victoria, Australia; Department of Obstetrics and Gynaecology, Monash University, Clayton, Victoria, Australia; Monash Data Future Institute, Monash University, Clayton, Victoria, Australia; City Fertility, Melbourne, Victoria, Australia

**Keywords:** artificial intelligence, machine learning, embryo, embryology, IVF, ART, embryo selection

## Abstract

**STUDY QUESTION:**

What is the present performance of artificial intelligence (AI) decision support during embryo selection compared to the standard embryo selection by embryologists?

**SUMMARY ANSWER:**

AI consistently outperformed the clinical teams in all the studies focused on embryo morphology and clinical outcome prediction during embryo selection assessment.

**WHAT IS KNOWN ALREADY:**

The ART success rate is ∼30%, with a worrying trend of increasing female age correlating with considerably worse results. As such, there have been ongoing efforts to address this low success rate through the development of new technologies. With the advent of AI, there is potential for machine learning to be applied in such a manner that areas limited by human subjectivity, such as embryo selection, can be enhanced through increased objectivity. Given the potential of AI to improve IVF success rates, it remains crucial to review the performance between AI and embryologists during embryo selection.

**STUDY DESIGN, SIZE, DURATION:**

The search was done across PubMed, EMBASE, Ovid Medline, and IEEE Xplore from 1 June 2005 up to and including 7 January 2022. Included articles were also restricted to those written in English. Search terms utilized across all databases for the study were: (‘Artificial intelligence’ OR ‘Machine Learning’ OR ‘Deep learning’ OR ‘Neural network’) AND (‘IVF’ OR ‘*in vitro* fertili*’ OR ‘assisted reproductive techn*’ OR ‘embryo’), where the character ‘*’ refers the search engine to include any auto completion of the search term.

**PARTICIPANTS/MATERIALS, SETTING, METHODS:**

A literature search was conducted for literature relating to AI applications to IVF. Primary outcomes of interest were accuracy, sensitivity, and specificity of the embryo morphology grade assessments and the likelihood of clinical outcomes, such as clinical pregnancy after IVF treatments. Risk of bias was assessed using the Modified Down and Black Checklist.

**MAIN RESULTS AND THE ROLE OF CHANCE:**

Twenty articles were included in this review. There was no specific embryo assessment day across the studies—Day 1 until Day 5/6 of embryo development was investigated. The types of input for training AI algorithms were images and time-lapse (10/20), clinical information (6/20), and both images and clinical information (4/20). Each AI model demonstrated promise when compared to an embryologist’s visual assessment. On average, the models predicted the likelihood of successful clinical pregnancy with greater accuracy than clinical embryologists, signifying greater reliability when compared to human prediction. The AI models performed at a median accuracy of 75.5% (range 59–94%) on predicting embryo morphology grade. The correct prediction (Ground Truth) was defined through the use of embryo images according to post embryologists’ assessment following local respective guidelines. Using blind test datasets, the embryologists’ accuracy prediction was 65.4% (range 47–75%) with the same ground truth provided by the original local respective assessment. Similarly, AI models had a median accuracy of 77.8% (range 68–90%) in predicting clinical pregnancy through the use of patient clinical treatment information compared to 64% (range 58–76%) when performed by embryologists. When both images/time-lapse and clinical information inputs were combined, the median accuracy by the AI models was higher at 81.5% (range 67–98%), while clinical embryologists had a median accuracy of 51% (range 43–59%).

**LIMITATIONS, REASONS FOR CAUTION:**

The findings of this review are based on studies that have not been prospectively evaluated in a clinical setting. Additionally, a fair comparison of all the studies were deemed unfeasible owing to the heterogeneity of the studies, development of the AI models, database employed and the study design and quality.

**WIDER IMPLICATIONS OF THE FINDINGS:**

AI provides considerable promise to the IVF field and embryo selection. However, there needs to be a shift in developers’ perception of the clinical outcome from successful implantation towards ongoing pregnancy or live birth. Additionally, existing models focus on locally generated databases and many lack external validation.

**STUDY FUNDING/COMPETING INTERESTS:**

This study was funded by Monash Data Future Institute. All authors have no conflicts of interest to declare.

**REGISTRATION NUMBER:**

CRD42021256333

WHAT DOES THIS MEAN FOR PATIENTS? IVF requires the fertilization of multiple eggs (oocytes), which over a few days develop into embryos that could be transferred to the patient’s uterus. At present, medical treatments for infertility, such as IVF, possess low success rates with most of the transferred embryos failing to implant in the uterus, and these problems increase in older women. One reason for this low success rate may be the embryo selection process, which involves a visual analysis by embryologists to select the embryo considered to have the best chance of resulting in a live birth. Success may therefore be, at least in part, dependent on the embryologist’s analysis of each embryo, which is prone to human error and variability because of individual subjectivity. The application of artificial intelligence (AI) could reduce this subjectivity and we carried out a review to explore the use of AI for embryo selection in IVF and compared it to various methods used by embryologists. The studies performed so far indicate that AI may provide more objective and accurate embryo quality assessments than trained embryologists. However, the application of AI in clinical care will first require further clinical validation in future studies. Additionally, in order to improve clinical relevance, there needs to be a shift in focus by AI developers for the output of AI, from a prediction of implantation likelihood to prediction of an ongoing pregnancy. If further studies confirm the current findings, AI may be a more reliable methodology of embryo assessment that can increase the likelihood of a healthy live birth for couples undergoing assisted reproduction.

## Introduction

Infertility, defined as the absence of conception after 1 year of unprotected intercourse (WHO, 2022), affects one to six couples trying to conceive during their life time (Njagi *et al.*, 2023), causing considerable emotional and financial distress (Gnoth *et al.*, 2003; Herbert *et al.*, 2009; Inhorn and Patrizio, 2015). Annually, the estimated number of couples experiencing infertility worldwide is around 17.5% of the adult population (one in six) (Njagi *et al.*, 2023).

As part of the treatment process, IVF requires the fertilization of multiple oocytes. Each oocyte is inseminated simultaneously under the same conditions and, if successful, eventually develops into an embryo that can be transferred into the patient’s uterus. Though touted as a cure for infertility, the success rate of ART procedures does not exceed 30% (Kanakasabapathy *et al.*, 2019). Indeed, for women above the age of 42 years, this rate can be <4% (Smith *et al.*, 2015). This low efficiency of ART success rate has been partly attributed to embryo selection assessments (Simon and Laufer, 2012; Bashiri *et al.*, 2018; Fishel *et al.*, 2020), as the majority of transferred embryos fail to implant in the uterus—a phenomenon which increases with female age (Bashiri *et al.*, 2018). Under current practice, embryo selection involves a visual analysis by embryologists to select the most viable embryo for implantation, while discarding poor-quality and slow-growing embryos. Success is therefore dependent on the embryologist’s analysis of each embryo, which is prone to human error and variability due to the intrinsic subjectivity of the process (Baxter Bendus *et al.*, 2006; Choucair *et al.*, 2021; Klimczak *et al.*, 2021; Geampana and Perrotta, 2023). Considering the high probability of treatment failure, patients often undergo five or more ART cycles to achieve a successful live birth (Smith *et al.*, 2015), leading to a high economic and emotional cost (Wu *et al.*, 2013; Drazba *et al.*, 2014; Mounce *et al.*, 2022). As such, there is a clinical need for a more reliable methodology of embryo assessment that can increase the likelihood of successful healthy live birth for couples undergoing ART treatments.

Recently, there has been a growing interest in applying artificial intelligence (AI) in ART, particularly during embryo selection (Kragh and Karstoft, 2021), treatment planning (Chow *et al.*, 2021), quality assurance in ART laboratories (Fernandez *et al.*, 2020), and sperm selection in ICSI (Mendizabal-Ruiz *et al.*, 2022). Indeed, AI in healthcare is a developing field that has demonstrated considerable promise, with successful applications in radiology (Hirsch *et al.*, 2022), genomics, and pharmaceuticals (Dias and Torkamani, 2019; Savage, 2021). In the case of embryo selection, AI could reduce the subjectivity associated with the embryo selection process by providing an accurate and reliable interpretation of embryo development status, embryo morphology, and potentially predicting embryo viability (VerMilyea *et al.*, 2020). However, owing to the niche technology introduced by AI, there are no current standards and expectations for its performance. Therefore, there are many questions regarding the methodology for comparing vital performance metrics, especially regarding generalizability and precision (Alipour and Harris, 2020). This is currently due to basic AI requirements, such as data quality and variety and large sample size, which can be resolved by the utilization of discarded embryos (de Hond *et al.*, 2022). Furthermore, the introduction of AI technology in IVF is in its infancy, including commercial AI systems already in clinical use where most of these factors are not being considered in a standardized manner. Therefore, this will likely result in different performances, particularly when models are compared against embryologists or in new sets of data. This has influenced the diffusion of AI in the IVF field in development phase without any prospective verification against embryologists using real-world data. It is also essential to analyse the inter- and intra-cohort embryo-assessment to precisely accommodate the application of the AI-model. Although unbiased samples are useful for the broader population, better predictions specific within the local culture can be more beneficial (Sundvall *et al.*, 2013).

Considering the current state of knowledge of AI in the setting of ART and the early stages of clinical implementation, this systematic review aims to provide an overview of AI application and AI techniques in the field of embryo selection, identifying the performance of these systems against the current embryologists’ assessments for both embryo morphology and reproductive outcome predictions. In doing so, we hope to raise awareness of the current landscape and discuss possible directions to inform AI developers, IVF clinicians, embryologists, and patients alike of the clinical and technical considerations that can facilitate clinical uptake.

## Materials and methods

This systematic review was conducted in accordance with the Preferred Reporting Items for Systematic Reviews and Meta-Analyses (PRISMA) guidelines (Page *et al.*, 2021). The study protocol and review were registered with the International Prospective Register of Systematic Reviews (PROSPERO) database (CRD42021256333).

### Search strategy and selection criteria

A systematic search was undertaken independently by two review authors (R.W. and M.S.) across PubMed, EMBASE, Ovid Medline, and IEEE Xplore from 1 June 2005 up to and including 7 January 2022. This date restriction was selected to ensure that only relevant technologies that reflect the present AI landscape were identified and to prevent outdated models that employed principles from the early days of AI from adding confounding factors that could influence the study conclusions. Included articles were also restricted to those written in English. Search terms utilized across all databases for the study were: (‘Artificial intelligence’ OR ‘Machine Learning’ OR ‘Deep learning’ OR ‘Neural network’) AND (‘IVF’ OR ‘*in vitro* fertili*’ OR ‘assisted reproductive techn*’ OR ‘embryo’), where the character ‘*’ refers the search engine to include any auto completion of the search term.

The inclusion criteria involved papers that had an application of AI in the field of embryo selection and were compared to at least one embryologist (user input); time-lapse or image segmentation technologies. Any other form of studies or procedure beside embryo selection were excluded. Articles from reference lists of studies being screened were also considered suitable for assessment. Additionally, articles focused on other fields of ART besides embryo selection (e.g. sperm and oocyte selection, controlled ovarian stimulation), did not contrast AI performance against an embryologist, focused on ultrasound, oncology, or non-human models, or focused on signal processing were excluded.

Articles were independently screened by M.S., M.M., and R.W. for suitability of inclusion and a list of eligible articles was created using Covidence^®^, a tool used primarily for screening and data extraction for Cochrane authors conducting standard intervention reviews efficiently by uploading search results, and screening abstracts and full-text study reports (VH Innovation, 2022). Following this, the full-text articles were read thoroughly by M.S., C.T., M.M., R.W., and F.H. Inclusion of an article for the review was based on consensus between these authors. Studies that did not relate to AI techniques with IVF were excluded.

### Quality assessment

Quality assessment was independently performed by two authors (M.M. and M.S.) using the Modified Down and Black checklist (Aubut *et al.*, 2013) (Supplementary Fig. S1). A third assessor, C.A., was involved in the event where a consensus could not be reached. This checklist provides scores for the quality of reporting, internal validity, and external validity, and can be applied to a broad range of study designs including non-traditional designs such as AI validation studies (Adom *et al.*, 2017).

### Summary of measures and synthesis of results

Data were extracted manually through the full-text review for analysis by three independent authors (M.S., C.T., and M.M.). The summary measures were presented in tabular form in this systematic review. Importance was given to the AI techniques implemented in the studies as well as their mode of classification for clinical interpretation (images/time-lapse videos, clinical data, or both) and performance (accuracy, sensitivity, and specificity) (Hossin and Sulaiman, 2015).

### Data analysis

For all machine learning techniques investigated, the medians and interquartile ranges (IQR) of accuracies were calculated to determine a summary accuracy between methodologies. Similarly, accuracies determined by clinical embryologists were also compared through medians and IQR. All other variables were expressed as a percentage. Owing to study heterogeneity, variations in AI techniques (in terms of inputs, output, the architecture of the AI, and the application of the AI), number of embryologists tested against for external validation, and accuracy as a performance metric, the performance of a meta-analysis was not considered appropriate.

## Results

**Figure 1.**
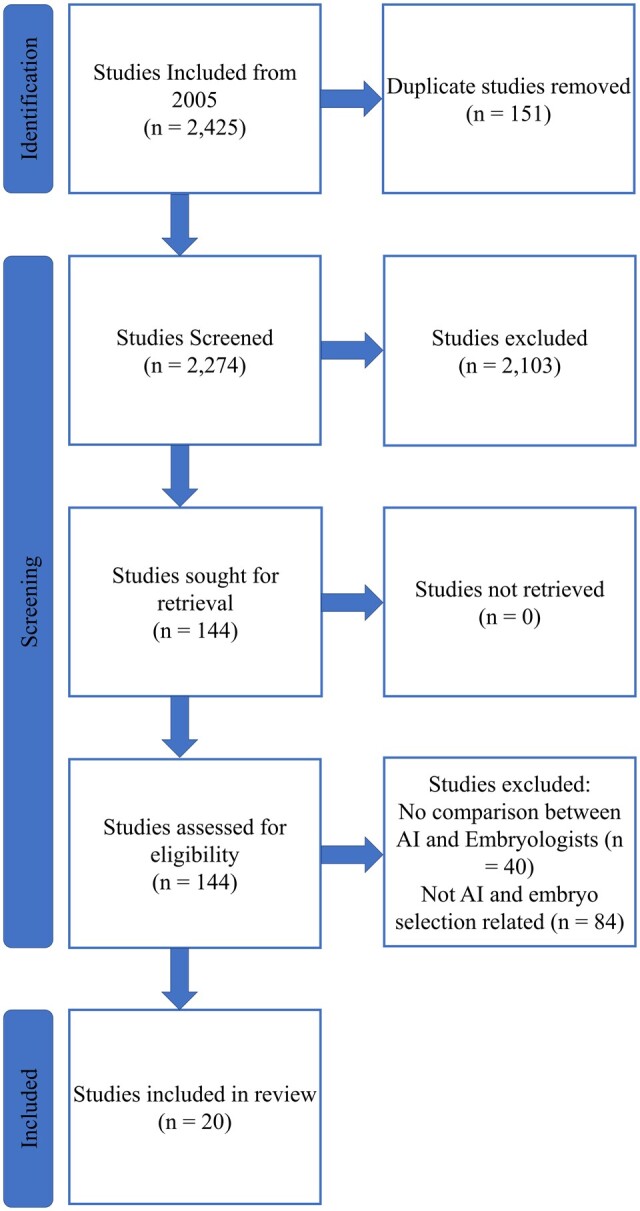
**PRISMA flow diagram of study selection for a systematic review of embryo selection through artificial intelligence versus embryologists.** The literature search included studies published since 2005. AI, artificial intelligence.

A total of 20 studies were included in this review. The article selection process is outlined in Fig. 1. The characteristics of the models included in this review are reported in Table 1. All the articles included in this review compared the performance of the AI against an embryologist or clinician. Each study had developed its own customized database from local patient populations making the ground truth subjective to the respective study. Each database had a different sample size, ranging from 224 samples to 14 144 samples. There was no specific embryo assessment day across the studies—Day 1 until Day 5/6 of embryo development was investigated. In addition, any reference to patient clinical data (e.g. age, BMI, etc.) has been referred to as clinical information throughout the review.

**Table 1.** Characteristics of studies included in a systematic review of embryo selection by using artificial intelligence or embryologists.

Author	Day of assessment	Number of embryologists	Technology	AUC	AI technique	Output
**Embryo assessment at blastocyst stage**						
Chavez-Badiola *et al.* (2020)	Day 5/6	2	Image segmentation	79	Deep neural network	Embryo aneuploidy
VerMilyea *et al.* (2020)	Day 5	2	Image segmentation	64	ResNet	Embryo morphology
Kragh *et al.* (2019)	Day 5	46	Time-lapse	69	Convolutional neural network	Embryo morphology
Rad *et al.* (2019)	Implantation day	1	Image segmentation	71	DenseNet—ResNet	Clinical pregnancy
Bormann *et al.* (2020a)	Day 5	15	Time-lapse	90	Convolutional neural network	Embryo morphology
Blank *et al.* (2019)	Day 5	1	Clinical record	74	Random forest	Clinical pregnancy
Bormann *et al.* (2020b)	Blastocyst day	10	Clinical record	52	Convolutional neural network	Embryo morphology
Khosravi *et al.* (2019)	Day 5	5	Time-lapse	98	GoogLeNet Inception V1	Embryo morphology
Loewke *et al.* (2022)	Day 5	Present—no number	Image segmentation	75	3× ResNet	Embryo morphology—ongoing pregnancy
**Embryo assessment at cleavage stage**						
Rad *et al.* (2018)	Day 1–3	Present—no number	Image segmentation	94	UNET	Embryo morphology
Coticchio *et al.* (2021)	Day 2	1	Image segmentation	83	LSTM	Embryo morphology
Bormann *et al*. (2021)	Day 3	7	Image segmentation	59	Convolutional neural network	Embryo morphology
Wu *et al.* (2021)	Day 3	4	Image segmentation	74	Convolutional neural network	Embryo morphology
Liao *et al.* (2021)	Day 1–6	4	Time-lapse	72	DenseNet201+ Focal loss + LSTM	Embryo morphology
Uyar *et al.* (2009)	Day 2–3	Present—no number	Clinical record	71	Support vector machine	Clinical pregnancy
Uyar *et al.* (2015)	Day 2–3	5	Clinical record	80	Naive Bayes classifier, k-nearest neighbour, decision tree, support vector machines, multilayer perceptron, and radial basis function network.	Clinical pregnancy
Hariton *et al.* (2021)	Day 1–2	1	Clinical record	68	Decision tree (light gradient-boosted machine)	Clinical pregnancy
Raef *et al.* (2020)	N/A	14	Clinical record	90	Support vector machine	Clinical pregnancy
**Embryo assessment at cleavage and blastocyst stage**						
Patil *et al.* (2019)	Day 2–5	1	Clinical record	86	Convolutional Neural Network	Clinical pregnancy
Sawada *et al.* (2021)	Day 1–6	Present—no number	Time-lapse	67	ResNet + attention network	Live birth

Day of assessment: day of embryo development when images or time-lapse videos were used to train artificial intelligence (AI) models; image segmentation: image processing techniques used to facilitate training of AI models; time-lapse: images captured over a period (usually days); clinical records: patient information used to train AI models; AI technologies: machine learning techniques and/or pre-trained AI models employed by included study; ResNet, residual neural network; DenseNet, dense neural network; LSTM, long short term memory; ICM, inner cell mass; TE, Trophectoderm. Accuracies were reported by studies in the form of a number between zero and one (Percentage), the numbers were converted to a whole number for tabulation purposes. No confidence intervals or *P*-values were reported.

Note: Any grade that contained B− or C and an extension rate equal to or less than three was considered part of the poor-quality group. In addition, any score with two A or A− grades, or one A with B, with an extension of three or greater could be labelled as good-quality. Some scores (e.g. 3BB, 3BA−) were debatable thus putting them in a separate category (fair-quality).

### Risk of bias assessment

Only three studies out of 20 scored greater than 7 out of 10 points on the Down and Black bias assessment tool, primarily due to the method of acquiring samples representing the population of their local country, which would signify a lack of generalizability if the AI were to be applied in another region or ethnic group (Supplementary Fig. S1). Despite so few studies scoring >70%, all studies were included in the analyses of this review. This is expected since this checklist was created with the intent of analysing clinical studies rather than preclinical AI validation studies. However, given the generalizability of this tool, it still provides a reasonable assessment of bias for the included studies.

### Models

In terms of the various networks used by each study, Table 1 shows the methods decided by the purpose and application of each study. For embryo images, variants of image-based convolutional neural networks (CNN) were utilized. CNNs use multiple layers of small filters passed over the entire image to identify features and make predictions. The variants used were: ResNet (Residual Network), which includes skip connections which allows the network to converge more quickly, while stabilizing the training (He *et al.*, 2016); DenseNet (Dense Network), which has extra connections to improve the network’s efficiency (Huang *et al.*, 2017); LSTM (Long Short Term Memory); and a time series model which can ‘remember’ information (Staudemeyer and Morris, 2019).

On the other hand, clinical information studies relied on vectorization and linear models: support vector machine, which linearly separates samples in groups for prediction (Hearst *et al.*, 1998); random forest and decision trees, a branching tree structure that uses data features to make a binary decision at each branch until it reaches an output (Bernard *et al.*, 2009), multilayer perceptron, which unlike the previously described clinical models is a neural network, and can have multiple layers of fully connected neurons (Murtagh, 1991).

### Synthesis of results

**Figure 2.**
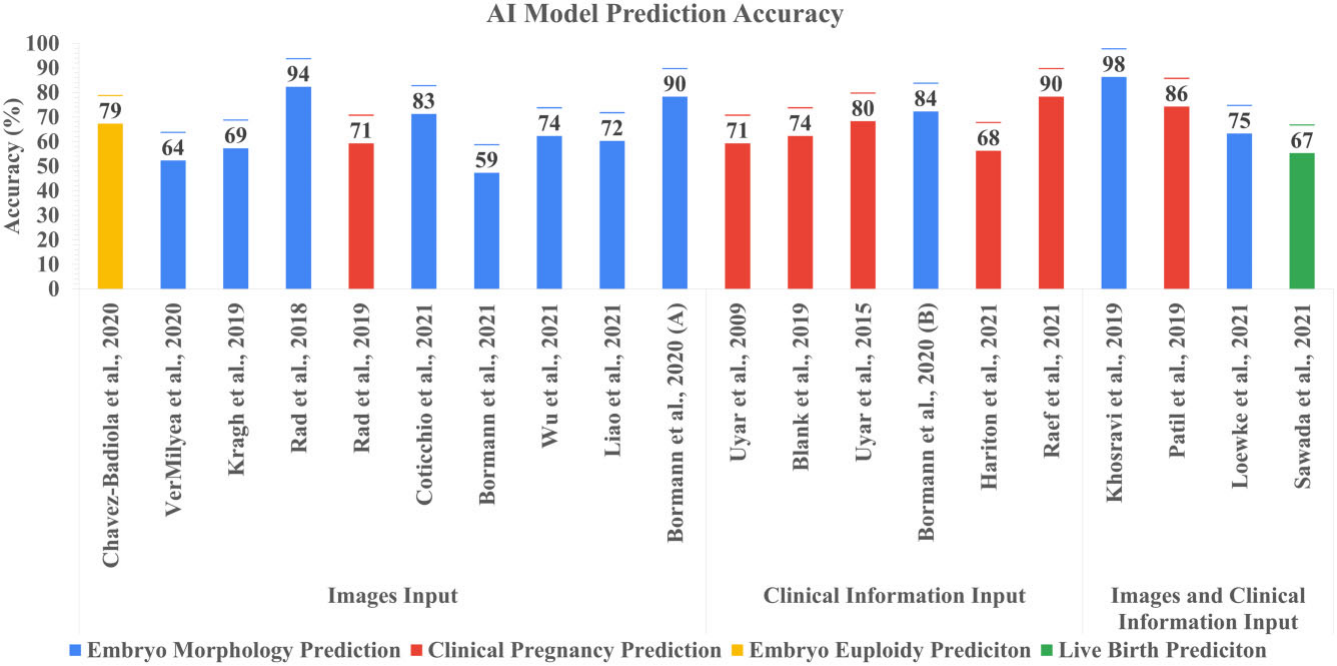
**Accuracy of the AI model defined by the data input utilized for model training.** Images input: studies including still embryo images and embryo images from timelapse videos. Clinical information input: studies including patient information features, demographics, and treatment information. Images and clinical information input: studies including the use of both embryo images and clinical information. The graph shows the accuracy output of the prediction defined by each study’s input sample type, such as embryo morphology prediction. Embryo grade though images and their quality assessments; clinical pregnancy prediction: prediction of possible successful clinical pregnancy. Embryo aneuploidy prediction: embryo aneuploidy prediction through embryo images. Live birth prediction: prediction of possible successful healthy live birth delivery. AI, artificial intelligence.

Sensitivity and specificity were reported only in three out of 20 studies (Uyar *et al.*, 2015; Chavez-Badiola *et al.*, 2020; VerMilyea *et al.*, 2020), while accuracy was the main metric reported in all studies. Figure 2 shows the accuracy reported by the studies, categorized by the type of input. The images category presented models which used images and time-lapses of embryos as inputs, typically resulting in an output of embryo morphological grade; clinical information represented patient information (often demographic data and clinical history) as an input to the models, with mainly clinical pregnancy prediction as the output. It is important to note that the accuracies reported for images only had slightly different outcomes as each AI had a different embryo morphology output (e.g. cell count versus cell features versus viability versus grade). This lack of standardization does not permit the comparison of models with each other. The lack of standardization was also inherited from the lack of ground truth. Indeed, the AI models used embryo images assessed following the specific grading guidelines of each clinic involved, while embryologist used the datasets following the same ground truth provided to the respective AI models. Hence, there was no common trend between the databases to create unionized inputs and outputs. Finally, four studies presented a mixture of embryo features combined with clinical information as inputs. Table 2 shows the sample size used by the presented studies. Each study developed its own database for AI validation; however, this was reported by only five out of the 20 reported studies (25%).

**Table 2.** Sample size of each study in a systematic review of embryo selection methods, including training, internal validation, and test set.

Author	Sample size (input to AI)	Training set	Validation set	Test set
Chavez-Badiola *et al.* (2020)	1231 entire dataset—840 used for AI	756	756	84
VerMilyea *et al.* (2020)	3604 pivotal (5282 pilot)	1744 (3892)	193 (390)	1667 (1000)
Kragh *et al.* (2019)	8664	6962	851	851
Rad *et al.* (2018)	224	190	Not available	34
Rad *et al.* (2019)	578 (8515)	492	Not available	86
Coticchio *et al.* (2021)	230 embryos	161	Not available	69
Bormann *et al.* (2021)	2449	1190	511	748
Wu *et al.* (2021)	3601	3601	Not available	699
Liao *et al.* (2021)	10 432 videos (577 cell counting model)	8346 (463 cell counting)	Not available	2086 (114 cell counting)
Bormann *et al.* (2020a)	2440 images	1188 (281 used for implantation training)	510	742 (29 used for implantation training)
Uyar *et al.* (2009)	2453	two-thirds	Not available	one-third
Blank *et al.* (2019)	1052	180 positive and 180 negative	Not available	73 positives and 231 negatives
Uyar *et al.* (2015)	3898	two-thirds	Not available	one-third
Bormann *et al.* (2020b)	3469	Not available	Not available	Not available
Hariton *et al.* (2021)	11 475	75% each patient	Not available	25% each patient
Raef *et al.* (2020)	1360	Not available	Not available	Not available
Khosravi *et al.* (2019)	12 001	10 075	Not available	1926 (964 good-quality images from 141 embryos and 966 poor-quality images from 142 embryos)
Patil *et al.* (2019)	535	428	Not available	images from 142 embryos
Loewke *et al.* (2022)	5923	All but 1000 images, 3-fold cross-validation	Not available	1000 images from one site
Sawada *et al.* (2021)	141 444	Not available	Not available	Not available

AI, artificial intelligence.

### Images and time-lapse

Ten out of 20 studies (50%) performed the classification with a solely image-based input system (Rad *et al.*, 2018, 2019; Kragh *et al.*, 2019; Chavez-Badiola *et al.*, 2020; VerMilyea *et al.*, 2020; Bormann *et al.*, 2020a, 2021; Coticchio *et al.*, 2021; Liao *et al.*, 2021; Wu *et al.*, 2021). These studies used a CNN as a backbone to create predictions. Only one out of the 10 studies (10%) used time-lapse as an input (Kragh *et al.*, 2019), by further employing a recurrent neural network. Rad *et al.* (2018) produced an accuracy of 93.8% on cell counting of embryo images, whilst Coticchio *et al.* (2021) scored 82.6% accuracy in applying a binary classifier to determine the ability of an embryo to reach the blastocyst stage.

### Clinical records and information

Six out of 20 studies (30%) focused on the use of clinical records (both patient and embryo-related clinical information) as an input to their models for predicting successful pregnancy (Uyar *et al.*, 2009, 2015; Blank *et al.*, 2019; Raef *et al.*, 2020; Bormann *et al.*, 2020b; Hariton *et al.*, 2021) except for (Bormann *et al.*, 2020b) where embryo morphology was the output of the model. Table 3 shows the clinical features used by each study as inputs to their respective models, where the female age can be seen as the common feature across all models and the embryo development stage the common embryo morphology feature. Bormann *et al.* (2020b) used a CNN approach, whilst the other five studies tested algorithms using statistical models. Uyar *et al.* (2015) achieved an accuracy of 80.4% in classifying implantation outcomes.

**Table 3.** Reported clinical characteristics included as features in the AI models.

Author	Patient clinical characteristics	Embryo morphology features
Uyar *et al.* (2009)	Woman’s age*, Infertility factor, Treatment protocol, FSH dosage, Peak Estradiol level	Early cleavage morphology, early cleavage time, transfer day, number of cells, nucleus characteristics, fragmentation rate, equality of blastomeres
Blank *et al.* (2019)	Female age*, Male age, AMH Level, TSH Level, Gravidity, Abortus, Therapeutic Abortus, Parity, Indication: (Tubal Causes, Endometriosis, Ovarian Causes, Genetic Causes (female), Genetic Causes (male), Uterine Causes, Male Factor, Non-medical Causes, Cervical Causes, Repeated Miscarriage, Hypothalamic Dysfunction, Immunologic Causes, Oncological Causes, Unexplained), Ovarian Stimulation Protocol, Down-regulation Protocol, Days of Stimulation, Number of Attempt, Sperm Source, Sperm Collection Method, Sperm Volume, Sperm Concentration, Sperm Motility Rate, Number of Oocytes, Number of fertilized Cells, Fertilization Method, Number of Egg Cells	Day 2 (cell stage, fragmentation, cytoplasma, cell-specific size, cell size, nuclei, MNB, vacuoles), Day 3 (cell stage, fragmentation, cytoplasma, cell-specific size, cell size, vacuoles), Day 4 (cell stages, vacuoles), Day 5 (cell stage, ICM, TE)
Uyar *et al.* (2015)	Female age*, Gravidity, Infertility factor, Treatment protocol, Utilized sperm, Duration of stimulation (days), FSH, Peak E2 level, Endometrium thickness, Early cleavage inspection time, Early cleavage morphology	Number of cells, nucleus characteristics, fragmentation rate, equality of blastomeres, appearance of cytoplasm, thickness of zona pellucida, transfer day (Days 2–3)
Hariton *et al.* (2021)	Trigger day, Estradiol, BMI, Age at start of treatment, Total no. of follicles, Estradiol/follicles, Maximum follicle size, Eggs retrieved, Mature eggs retrieved, Total no. of eggs fertilized	Total usable blastocysts
Raef *et al.* (2020)	Female age*, male age*, BMI, Family relation of couples, Family relation in parents of couples, Smoking, Type of infertility, Infertility duration, Contraception duration, Infertility in family, gravida, parity, abortion, ectopic pregnancy, living children, dead children, Comorbidity diseases, Anemia, Thyroid disease, Prolactin hormone disorders, Drug usage, Amenorrhea, Dysmenorrhea, Period status, Hirsutism, Galactorrhea, Gynecological surgery, Oocyte donation, AFC, Endometrium thickness, Three-line (regular/normal) endometrium, Uterus depth, Size of follicles, Tubal factor, Pelvic factor, Cervical factor, Ovulatory factor, PCOS, Uterine factor, Endometriosis, Endometrial factor, Vaginitis, RIF, recurrent pregnancy loss, Thrombophilia disorders, Immunologic disorders, Male factor, Male genital surgery, Varicocele, TESE, PESE, Fresh/freeze sperm, Sperm count, Normal morph, Immotile, FSH, LH, Estradiol vitD3 Levels, FSH/HMG dosage, GnRH, antagonists Dosage, GnRH agonists dosage, Duration of stimulation (days), Estradiol dosage, No. estradiol days, Number of retrieved oocytes, Number of metaphase II quality oocytes, Number of metaphase I quality oocytes, Number of germinal vesicle quality oocytes, Number of degenerated quality oocytes, Quality of injected metaphase II oocytes	Number of 2PN (pronuclear), number of developed embryos, quality of developed embryos, quality of vitelline space, ET (embryo transfer), ET day, number of transferred embryos, number of blastomeres, quality and stages of transferred embryos, experience of ET
Sawada *et al.* (2021)	Age*, Method of fertilization, Type of culture media	Mode of embryo transfer

AI, artificial intelligence; RIF, repeat implant failure; PESE, percutaneous epididymal sperm extraction; TESE, testicular sperm extraction; AFC, antral follicle count; AMH, anti-Müllerian hormone; TSH, thyroid-stimulating hormone; E2, estradiol; vitD3, vitamin D3; ET, embryo transfer; 2PN, 2 pronuclei; ICM, inner cell mass; TE, trophectoderm; MNB, multinucleated blastomeres. (*) indicates the feature with most influence on the performance of the AI model.

### Integration of images and clinical information

Four out of 20 studies (20%) utilized a combination of clinical information and embryo images as an input to the AI models (Khosravi *et al.*, 2019; Patil *et al.*, 2019; Sawada *et al.*, 2021; Loewke *et al.*, 2022). All four studies utilized an open-source AI model (GoogleNet and ResNet—Table 1) to deliver accuracies of 97.5% (Khosravi *et al.*, 2019), 86% (Patil *et al.*, 2019), 75% (Loewke *et al.*, 2022), and 67% (Sawada *et al.*, 2021).

### AI against embryologist

**Figure 3.**
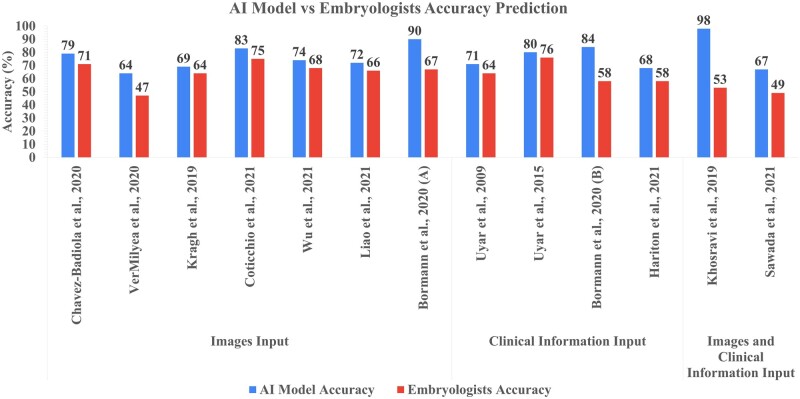
**AI model accuracy compared to prediction accuracy of embryologists according to type of data input.** Blue: AI model accuracies among studies. Red: embryologists accuracies among studies. Images input: studies including still embryo images and embryo images from timelapse videos. Clinical information input: studies including patient information features, demographics, and treatment information. Images and clinical information input: studies including the use of both embryo images and clinical information. AI, artificial intelligence.

When comparing the accuracies of AI models against embryologists, all the studies reported a better performance in favour of the AI models by at least 4% higher prediction accuracy. The greatest difference can be seen in Khosravi *et al.* (2019) where the AI outperformed the embryologists by 45% when correlating embryo quality with implantation outcome. Figure 3 shows the performance of embryologists when compared with the AI, when both are exposed to blind tests of data. The overall median accuracy of AI models was 74% (range 64–98%, IQR = 68.5–83.5) compared to embryologists' accuracy of 64% (range 53–76%, IQR = 55.5–69.5) (Uyar *et al.*, 2015; Khosravi *et al.*, 2019; VerMilyea *et al.*, 2020).

### Prediction analysis

**Figure 4.**
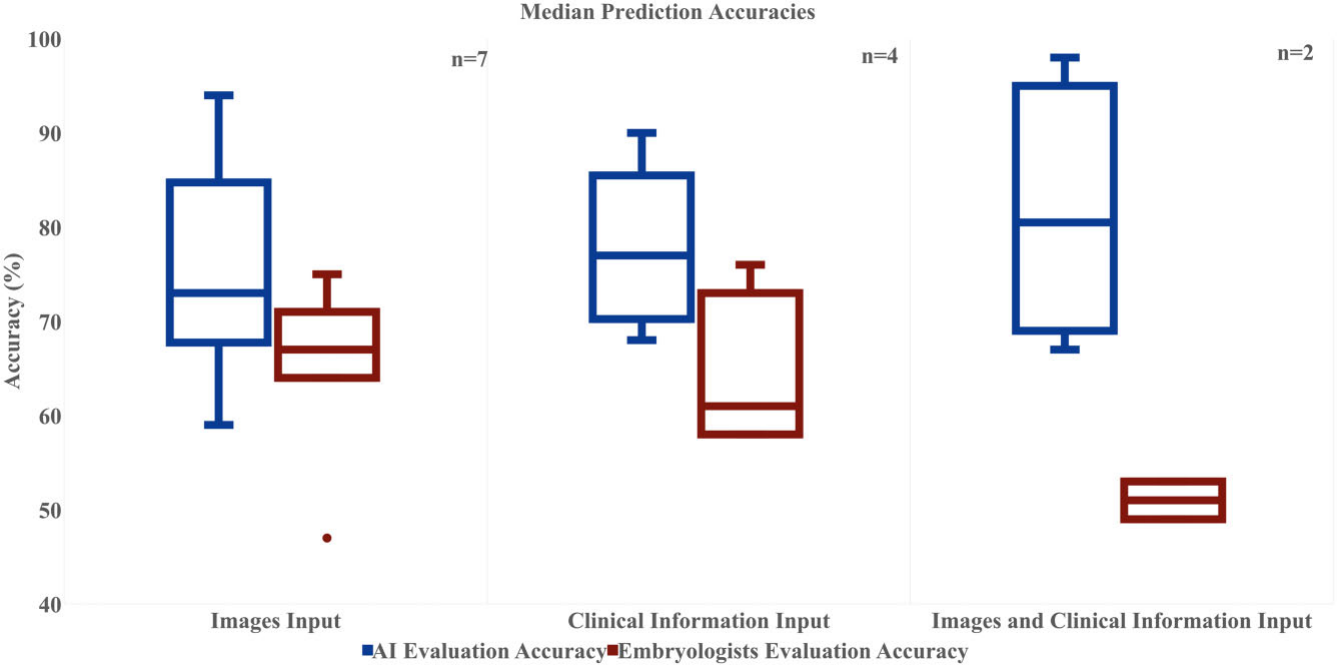
**Median accuracy and interquartile range for AI models compared to embryologists’ predictions.** Images input: studies including still embryo images and embryo images from timelapse videos. Clinical information input: studies including patient information features, demographics, and treatment information. Images and clinical information input: studies including the use of both embryo images and clinical information. Sample size (n) refers to number of studies on each analysis. AI, artificial intelligence.

The reported studies in this review fall under three categories, which are dependent on the type of input used: images, clinical information, and combined (images and clinical information). The median accuracy and IQRs are reported in Fig. 4, where the integration of both images and clinical information had the highest median accuracy (81.5%, IQR = 69–95%) compared to the embryologists (51%, IQR = 49–53%). Furthermore, AI models had an overall higher median accuracy; for image samples as an input, AI produced median accuracy of 75.5%, IQR = 59–94%, while embryologists scored 65.2%, IQR = 64–75%. A similar trend can be seen in clinical information as an input, where AI models produced median accuracy as an output of 77.8%, IQR = 68–90% while embryologists predictions output were 64%, IQR = 58–76%. As noted, all AI models also presented higher IQR for all type of predictions.

## Discussion

This systematic review discusses the present landscape of AI application studies for embryo selection. To do this, we have analysed the current literature to develop a comparison of AI performance against embryologists, as the key clinicians involved in embryo selection procedures. The current results show a higher accuracy of AI models against embryologists; however, there is a strong variability in the performance, indicating a necessity to establish what are the correct AI model features required for clinical implementation, as well as the minimum AI benchmark performance and correct datasets for validation in the IVF field. In comparison, when blind test samples are presented to both AI and embryologists, there was a consistency in AI outperforming the embryologists, whether data were embryo images or clinical information. The results also showed that the choice of AI is essential for the application performance. Moreover, the size of the sample base plays a key role in the number of layers of the AI and their size, which significantly varies among included studies. This indicates that there is no commonality between the various models in the current literature as well as current AI models used for clinical applications.

### Images and time-lapse

The ability of computer vision, and more specifically CNNs, to study an image pixel by pixel and recognize contextual patterns makes it highly useful in this application (Yamashita *et al.*, 2018). This enables the model to perform sufficiently in a clinical setting; however, it does have the setback of requiring considerable computational power for training (Willemink *et al.*, 2020). This power requirement is dependent on the size of the training dataset, where a considerable number of samples are required for the outputs of the AI to be generalizable to the broader population. For instance, Chavez-Badiola *et al.* (2020) developed ERICA, which produced a 79% accuracy compared to the two embryologists involved in the study (71% and 63%). Similarly, VerMilyea *et al.* (2020) scored a higher accuracy (64%) than the embryologist (47%).

Overall, CNN has been the primary choice for the studies included in this review. Rad *et al.* (2018, 2019), Khosravi *et al.* (2019), VerMilyea *et al.* (2020), Sawada *et al.* (2021) and Loewke *et al.* (2022) have all employed pre-trained networks (ResNet, DenseNet, and U-Net), which were then further trained on their datasets, reducing the total training time required. Pre-trained networks also provide a validated system as they are already proven to work on a more general dataset with near 100% accuracy (Dan Hendrycks *et al.*, 2019). The difference can be found in the output layers, which are configured for the different applications, especially for time-lapse samples, where 3D samples can also be provided as an input. In the case of time-lapse data, the samples are still broken down to single images dependent on the point in time under investigation; however, this will give the user the option to define the better image capture to utilize as the input. This reduces the need for the immense computational power necessary to interpret large time-lapse videos while preventing the compromise of its quality; hence the highest performing study focused on time-lapse (Rad *et al.*, 2018, 94%).

### Clinical records and information

Clinical information has been evaluated as an input to machine learning models. This is because clinical records can provide considerable amounts of information regarding the treatment and patient condition such as patient phenotype. Here, principal component analysis (PCA) would be more effective than machine learning techniques for considering the essential features to be used in the AI algorithm (Karamizadeh *et al.*, 2013). This is because the PCA technique finds the common trends within the information and helps to determine the relative importance of the variables based on their influence on the outcome, which is necessary given the multivariable nature of the information contained within clinical records of patients with infertility. Studying clinical records can sometimes introduce various variables that can be deemed insignificant to the AI model (Lee and Kim, 2021). Unlike AI, in clinical settings, the embryologists would also consider risks that are associated with treatments performed by the clinical team (Kragh and Karstoft, 2021). Hence, embryologists tend to make conservative, educated decisions (Petersen *et al.*, 2016).

Unlike images and time-lapse, clinical information lacks the ability to understand embryo quality. Uyar *et al.* (2015) found that the main two features of interest in clinical information are women’s age and serum FSH level. Whilst such clinical information is presently generalized to the broader population, it is distinct to the patient, signifying the potential for AI to enable a personalized prediction as individual patient information could facilitate tailored outputs according to patients’ clinical history. Although at present, AI models have not well defined the integration of clinical information, women’s age, and FSH are the preferred data points in AI classification of clinical pregnancy, implantation, and live birth (Goyal *et al.*, 2020). However, other features should not be neglected as there is a discernible trend whereby incorporating more features into the AI models could lead to more confident predictions (Pettit *et al.*, 2021). For example, Raef *et al.* (2020) performed the best, achieving 90% accuracy, when presented with the highest number of features as compared to the other studies included in this systematic review.

### Integration of images and clinical information

The idea of combining the clinical information and images into one AI model has the potential to provide a tailored approach that is specific to the patient and their needs, for a more personalized treatment. This is a direction that medical technologies are taking towards the future, as each patient has their own traits for which personalized approaches can play an important role (Thimbleby, 2013). The category of combining clinical information and characteristics with embryo images and time-lapse has demonstrated to be a valuable approach in providing the best support by achieving the highest accuracy in the selected studies in this systematic review (81.5%). Although the current studies include limited clinical information integrated into AI models, the incorporation of patients, and IVF treatment information into AI models could lead to the creation of various additional suggestions to prompt embryologists and clinicians to support clinical decisions at the time of embryo transfer. Incorporating this information into AI models could provide insights into the history behind patients’ infertility, which could have implications regarding the prognosis of patients. For instance, patients with poor prognosis could be identified by the number of ART cycles they have attempted as well as the presence of increased reproductive age in the couple, which is known to negatively impact live birth outcomes in ART (Horta *et al.*, 2019). The addition of different age brackets can help AI models to determine the output produced by patients of different ages and help the model generalize better on the overall sample size (de Hond *et al.*, 2022). Furthermore, under circumstances of different patient prognosis, such as advanced reproductive age, the traditional embryo selection criteria probably have a lower weight in the final reproductive outcomes, as for the vast majority of the embryos produced by those patients, embryo selection will not lead to an improved outcome (Sanchez *et al.*, 2019). However, it could be hypothesized that the number of embryos to transfer under some circumstances could increase the chance of achieving pregnancy, despite the known negative implications, particularly when only poor-quality embryos are present (Kemper *et al.*, 2021). Additional possibilities around embryo transfer include the decision to transfer fresh or frozen embryos, as this is also dependent on clinical information (Bushaqer *et al.*, 2020). Importantly, although the use of poor-quality embryos is controversial, they could be of significant value for patients with poor prognosis as the transfer of low-grade embryos still has the potential to achieve live birth (Kemper *et al.*, 2021). Thus, incorporating the patient information into AI models in the future could help to further develop clinical decision support tools tailored to the needs of each patient, primarily based on their prognosis. However, it is important to highlight that these systems will not be able to improve the efficacy of ART treatments in all cases. Nevertheless, information about patient prognosis could be used to counsel patients and potentially explore different reproductive alternatives.

### Clinical implications

All of the included studies have created their own datasets. This can result in datasets that do not adequately represent common practices or the broader population owing to limited access to data from different ethnic groups (Fremont *et al.*, 2016). It also makes it difficult to compare models as there is no similarity in datasets or intended prediction outcome (Lee and Lee, 2018). However, creating individual datasets can allow them to be relevant to the local population. This is, therefore, a suitable practice to avoid data shifting, where the older samples in the dataset become more negligible compared to the newer data, and provides relevant exposure to the present challenges in embryo features detection and embryo selection (Racowsky *et al.*, 2010). Hence, these databases provide sufficient samples to justify developing and testing modern machine learning techniques. The type of sample will define the appropriate model to be used; relevant patient information will require a small shallow model, while embryo images and time-lapse videos will require a deeper model to extract the useful features from the images. Another idea is combining both inputs (clinical information and embryo images), where the model can make a more confident prediction with the features extracted from the images and relate the features to the clinical patient information; therefore, a deeper model comprising both inputs combined will be preferred.

Another point that must be raised is that the method of quality assessment of the embryo was dependent on the researcher rather than utilizing a standardized grading system for the development of the AI as an input and output (ESHRE Special Interest Group of Embryology and Alpha Scientists in Reproductive Medicine, 2017; Puga-Torres *et al.*, 2017). Therefore, without a consensus for which grading system to use, the clinical relevance of these models is restricted to clinics that are willing to adopt a new grading system or already use the system employed by the AI model. Although the quality evaluation has been correlated to pregnancy outcome (Diakiw *et al.*, 2022; Park *et al.*, 2022), embryologist-defined quality evaluations do not necessarily correlate to the outcome of ongoing pregnancy (Zhao *et al.*, 2018; Kirillova *et al.*, 2020). This is particularly relevant as there is a high degree of subjectivity in embryo quality assessment (Fishel *et al.*, 2020; Bormann *et al.*, 2020b; Fordham *et al.*, 2022). A seemingly ‘poor’ embryo can still result in an ongoing pregnancy whilst a ‘good’ embryo may not result in successful implantation (Wintner *et al.*, 2017). Hence, the requirement of ground truth labelling and the inability to consistently do so in the different studies owing to the lack of standard definitions can limit the reliability and prediction capabilities of the AI model. However, the common use of successful implantation as an outcome measure is also a limiting factor for these AI models as the intended outcome of any IVF cycle is to produce a live birth. Finally, the current clinical practice of discarding ‘poor’ embryos is itself a limiting factor for AI development in the field as these embryos could possess value in training the machine learning algorithm by adding a stronger comparison class of samples (Simopoulou *et al.*, 2019).

Based on the findings of this review, AI has demonstrated the potential to predict embryo quality with high accuracy. However, none of these systems has been proved to provide predictions to the degree necessary for clinical implementation, as the training datasets are skewed by a lack of understanding of the complexity of the clinical challenge. So far, the models have only been tested retrospectively; thus, have not been validated clinically. As such, there is limited evidence that the models will perform at the same accuracy prospectively. Although the present validation is promising and justifies further technical development, more concrete clinical trials are still essential for considering if implementation is feasible.

### Limitations

The findings of this review should be interpreted with caution. Firstly, although the review discusses the involvement of embryologists in the validation of the developed technology, the included studies did not show any intent to take the developed technology to a clinical trial stage. The studies mentioned in this review stated that further research is required in applying AI in ART. Secondly, a fair comparison of all the studies is deemed unfeasible as each study tested on their own generated databases without an external verification step through the research centres associated with the team carrying out the study. Hence, the review has only considered the technique of AI used, in addition to the sample size and the performance of the embryologists consulted in each study. Finally, whilst the databases were interpreted correctly across all studies, differences in skill, experience, and training of the developer can influence the accuracy and reliability of the AI model and influence the decision of the embryologist. The lack of consistency between embryo stage and clinical features in the studies further constraints the ability to perform meta-analysis owing to heterogeneity between studies. Despite these limitations, it must be highlighted that the included studies have demonstrated promise and have shown potential application for the integration of AI as a decision-support tool in the field of IVF.

## Conclusion

The current findings on the application of AI for embryo selection indicates that AI models can outperform embryologists in terms of embryo morphology assessments as well as reproductive outcome predictions. However, it is important to highlight that the current accuracies reported by studies present different performances owing to the diversity of methods employed, sample size as well as the dataset used for AI training and AI validation. Thus, it is critical to establish the minimum AI models features required for clinical implementation as well as the AI benchmark performance and correct datasets for validation in the IVF field. The best performing prediction models for clinical outcomes combined clinical information and images, indicating that the use of clinical data could improve the performance of models that only rely on the use of images or time-lapse videos. Future studies should focus on the prediction of live birth, which is clinically the most relevant outcome of ART.

## Supplementary Material

hoad031_Supplementary_Data

## Data Availability

The data underlying this article are available in the article and in its online supplementary material.
