# Supplementary material for: Embryo selection through artificial intelligence versus embryologists: a systematic review
Source: Hum Reprod Open. 2023 Aug 15;2023(3):hoad031. doi: 10.1093/hropen/hoad031 (PMC10426717; doi:10.1093/hropen/hoad031)
Supplement: hoad031_Supplementary_Data [file hoad031_supplementary_data.pdf]

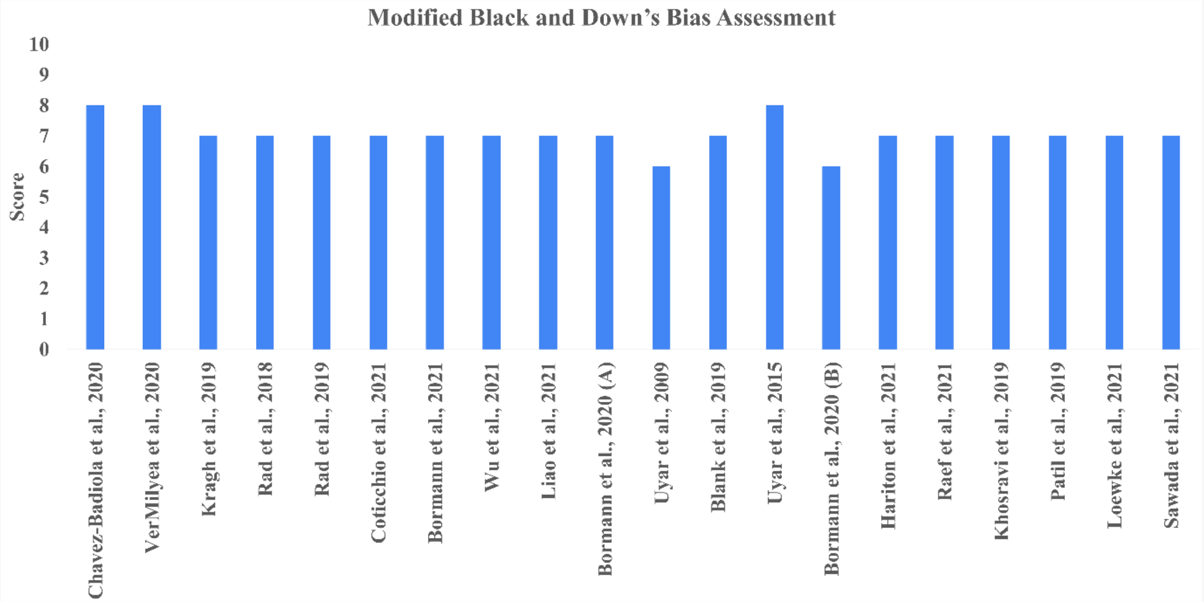

Supplementary Figure S1. Modified Black and Down's score for quality of studies included in review (Maximum score achievable is 10 points).
